# Analog time-reversed ultrasonically encoded light focusing inside scattering media with a 33,000× optical power gain

**DOI:** 10.1038/srep08896

**Published:** 2015-03-10

**Authors:** Cheng Ma, Xiao Xu, Lihong V. Wang

**Affiliations:** 1 Optical Imaging Laboratory, Department of Biomedical Engineering, Washington University in St. Louis, St. Louis, Missouri 63130-4899, USA

## Abstract

Recent breakthrough in wavefront engineering shows great promises in controlling light propagation inside scattering media. At present, the digital approaches enjoy high gain, while their speeds are slow because of high data throughputs. In contrast, the analog approaches are intrinsically fast but suffer from poor efficiencies and small gains. Further improvements in both speed and gain are necessary to advance the existing technologies toward real-world applications. Here, we report analog time-reversal of acousto-optically tagged photons with a flux amplification of over 33,000 times (45 dB) at a target location inside scattering media. Such a substantial power gain enhancement is achieved when the temporal width of the time-reversed photon packet is squeezed below the carrier-recombination-limited hologram decay time in a photorefractive crystal. Despite a focusing energy gain below unity, the unprecedented power gain is expected to enable new optical imaging, sensing, manipulation and treatment applications.

In the presence of micro-scale heterogeneity, scattering impedes our ability to control light for imaging, sensing, machining, treatment and manipulation. As a prominent example, biological tissue—due to its optically random and complex structure—scatters light so strongly that 1 mm-thick tissue renders the ballistic (i.e., unscattered) component of light negligible^1^,^2^. A fundamental problem is to focus light in the diffusive regime^2^,^3^. By taking advantage of the time-reversal symmetry of the electromagnetic wave equation for lossless media, scattering compensation boils down to determining the wavefront emitted by a “guide star” located at the targeted focal position and performing a subsequent time reversal^4^, which is commonly realized via phase-conjugation of monochromatic light^5^. The desired wavefront can be found either by characterizing a subset of the transmission matrix element-wise^6^,^7^,^8^,^9^,^10^ or by a direct holographic measurement at once^5^. While the former approach is used in wavefront shaping (WFS) with simpler experimental configurations, the latter is used in direct time reversal for potentially faster responses. For *in vivo* applications in biological tissue, a response time on the order of 1 ms is desired due to fast speckle decorrelation^11^, and the higher speed of direct time reversal makes it more appealing.

Time-reversed ultrasonically encoded (TRUE) light focusing^12^ provides a viable means for direct time reversal, in which an ultrasonic focus is employed as a virtual guide star. TRUE focusing can be implemented with digital or analog phase conjugate mirrors (PCM). In the digital version, a spatial light modulator is used as the PCM^13^,^14^. The upper limit of the focal peak-to-background ratio (PBR) is^13^ (*π*/4)(*N* + 1)/*M*, where *N* is the number of controlled degrees of freedom (DOF), and *M* is the number of speckle grains within the focus. Because *N* is ultimately limited by the SLM's pixel count and *M* ranges from 10^3^ to 10^5^ depending on the ultrasonic frequency, the achievable PBR is low (typically below 10)^13^,^14^. Moreover, since the speed is limited by the rates of data transfer, data processing, and SLM actuation, state-of-the-art systems run well below 1 Hz.

In analog TRUE focusing, optical phase conjugation is performed by a photorefractive crystal (PRC)^12^, which can accommodate a higher DOF (at least two orders of magnitude more than that of digital PCMs^15^), and is potentially capable of responding faster than 1 ms^16^. The light beams participating in hologram recording and readout are coherent, derived from a single continuous wave (CW) source. The gain of the focusing procedure, defined as *G*_p_ = *P*_TR_/*P*_S_, where *P*_TR_ and *P*_S_ are the powers in the focal volume during time reversal and hologram recording respectively, is much less than unity^13^ due to the low energy conversion efficiency. That is, the energy gain *G*_E_ = *E*_TR_/*E*_S_, defined through the energy ratio between the time-reversed and the sample beams in the focal volume, is much less than unity. The application of the analog approach is primarily hindered by the low gain.

For a fixed number of incident photons, the signal-to-noise ratio (SNR) of optical detection follows (see Supplementary Information)
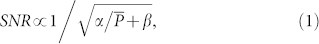
where *α*, *β* are constants, and 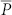 denotes the average optical power. Since the diffuse light intensity experiences an exponential decay versus depth in scattering media, signals coming from deeper regions may easily fall below the detection limit of the photodetector. According to Eq. (1), a sufficiently large power gain can improve the SNR and render the originally invisible signal readily detectable. In addition, an optical power enhancement can dramatically increase nonlinear optical signals^17^ and intensify the radiation force for optical manipulation^18^. Consequently, a power gain much greater than unity is indispensable to a number of scattering-limited applications.

In this letter, we demonstrate an analog TRUE focusing system with an unprecedented power gain of 33,000 times (45 dB), approaching that achieved by the digital configurations^13^. The key to this extraordinary gain is a protocol in which a low power quasi-CW beam and a high power pulsed beam are enabled in the hologram writing and reading processes, respectively. A similar scheme was previously explored using a BaTiO_3_ crystal for image amplification with large two-wave mixing (TWM) gain and reduced fanning noise^19^. The pursuit of such a high gain entails a detailed understanding of the energy conversion efficiencies in the focusing procedure as a prerequisite, and is challenged and complicated by the PCM's transient behaviors under high-intensity illumination.

A simplified rendering of the system set-up is shown in Fig. 1 (a detailed schematic is given in the Methods). All light beams are in the ***x***-***y*** plane, while ultrasound is applied along –***z***. A 532 nm solid state laser (CNI, PGL-532) with a CW output power of 50 mW is used for hologram writing. The CW beam is split into a reference beam R and a signal beam S by a polarizing beamsplitter (PBS) with a splitting ratio adjustable via a half-wave plate (HWP). The signal beam interferes with the reference beam in a photorefractive Bi_12_SiO_20_ (BSO) crystal (1 × 1 × 1 cm^3^), where the interbeam angle is 13 degrees for optimum diffraction efficiency. The crystal is oriented so that the entrance face is a (110) plane with the surface normal equally bisecting the beam angle. Frequency shifts *f*_1_ and *f*_2_ are introduced in R and S by acousto-optic modulators AOM1 and AOM2 (505AF1, IntraAction), respectively. R is broadened by an afocal beam expander BE1 to a diameter of 1 cm before impinging on the PRC. While S illuminates the scattering medium (SM), the scattered light, collected by a pair of lenses (L1 and L2), is concentrated on the PRC. The lens pair images the back surface of the SM onto to the entrance face of the PRC. The reading beam R* is generated by a separate, much stronger pulsed laser. Two types of pulsed lasers, generating 10 ns (Brilliant B, Quantel) and 5 ps (APL-4000, Attodyne) pulses at 532 nm, are used in the study. R* is broadened by an afocal beam expander BE2 to a diameter of 1 cm and aligned to be conjugated to R. Upon diffraction by the PRC, a portion of R* is converted to S* (the phase conjugate of S). The hologram writing and reading processes are controlled by switching on and off the writing beams (R and S) and the reading beam R*, by using a shutter ST (LS3Z2, Uniblitz) and AOM3 (505AF1, IntraAction), respectively.

The system was characterized by a “direct transmission” experiment, in which the scattering sample comprised two ground glass diffusers (DG10-600, Thorlabs) in series with a 2 mm gap in between. Since no ultrasonic tagging was involved, *f*_1_ = *f*_2_ = 50 MHz. Reading the hologram by R* generated S*, which back-traced S and became a plane wave after propagating backward through the scattering sample (point A in Fig. 1). A portion of the plane wave S* was directed to an energy meter (Vega, Ophir) by a 50:50 beamsplitter. This arrangement allowed us to directly determine that the energy gain *G*_E_ (the energy ratio between S* and S at point A in Fig. 1) was 10^−5^ when 350 μW sample beam power, 2 s hologram writing time, 200 μJ reading pulse energy and 10 ns pulse duration were used. If the duration of the reading pulse is long, it roughly takes the same amount of energy (~500 μJ cm^−2^) to write and erase a hologram in the PRC^20^, which sets the upper limit of *G*_E_ to be unity (among the available PRCs, BaTiO3 is distinguished by a very large electro-optic coefficient and is widely used for optical phase conjugation and image amplification. However, due to its slow response and large fanning noise, we chose to use a photorefractive BSO crystal in the demonstration). Moreover, the effective diffraction efficiency, defined as the energy of S* in the focus (for the “direct transmission” experiment, the energy of S* at point A in Fig. 1) divided by the energy of R*, can be expressed as
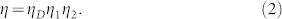
Here, the diffraction efficiency (the ratio between the diffracted and the reading pulse energies) *η_D_* = 0.1–1% typically^21^. The time-reversal efficiency (the ratio between the PRC-collected and the total scattered light) *η*_1 _≈ 10% in our case due to partial phase conjugation and reciprocity^22^. The wavefront reconstruction efficiency (the ratio between the energies of the correctly reconstructed and the entire diffracted light) *η*_2_ ≈ 1–10% in our case due to the imperfect PRC response in the Fourier domain, as analyzed below.

For simplicity, we neglect light polarization and consider only scalar fields. The sample and reference electrical fields are denoted as *E*_s_(**r**) and *E*_R_(**r**), respectively, where **r** denotes the spatial position. On the PRC input plane, the sample beam can be decomposed by the two-dimensional (2D) spatial Fourier transformation:
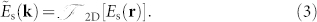


Here, 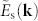 is the complex amplitude of the spatial harmonic component associated with wavevector **k**. This component interferes with R, whose wavevector and complex amplitude are **k**_R_ and 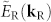, respectively, to generate a stable volumetric intensity fringe pattern in the PRC having a grating vector **K** = **k** − **k**_R_ (fulfilling the Bragg condition) and a complex amplitude 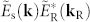. Due to the photorefractive effect^18^, the fringe induces in the PRC a dielectric constant modulation (a volume grating) of wavevetor **K** and complex amplitude
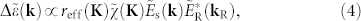
where *r*_eff_(**K**) is the effective electro-optic coefficient which is both real and **K**-dependent, and 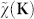 is a PRC-dependent complex parameter given by
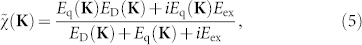
where *E*_ex_ is the externally applied field, *E*_q_(**K**) = *eN*_A_(1 − *N*_A_/*N*_D_)/(*ε*_s_|**K**|) and *E*_D_ = *k*_B_*T*|**K**|/*e*, with *N*_A_, *N*_D_ being the densities of the acceptors and donors, respectively, and *ε*_s_ is the static dielectric constant. During the hologram reading process, the above grating diffracts a portion of the reading beam toward −**k**. According to the coupled mode theory^21^, the diffracted component has a complex amplitude (below, we use the subscript “rec” to represent an imperfectly reconstructed field as opposed to the ideal conjugate beam 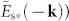)
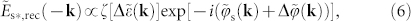
where *ζ*[*x*] is real and periodic with *x*, the phase 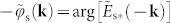 (arg[·] denotes taking the argument) implies phase conjugation, and 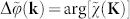 is a phase error (a **k**-independent constant phase shift is omitted).

The spatial domain reconstructed field S* can be readily obtained by an inverse 2D Fourier transformation performed on the input plane:
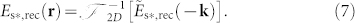


The wavefront reconstruction efficiency relative to the ideal conjugate field 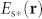 is found by an overlapping integral:
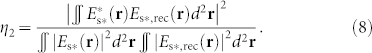


According to the Parseval's theorem, the above expression for *η*_2_ is equivalent to
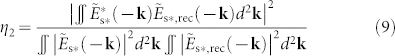


For regular or moderately distorted wavefronts, the magnitude of 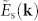 is appreciable only at narrowly distributed **k**; thus, according to Eq. (9), *η*_2_ can approach 100%. However, for diffuse light, 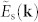 distributes over a broad range of **k**, and the value of *η*_2_ is always less than unity according to the Cauchy-Schwarz inequality.

According to the above analysis, *η* is determined once the experimental configuration is fixed. To overcome the efficiency barrier set by the hologram erasure, one can send in as many R* photons as possible within the hologram decay time. However, the real situation is far more intricate, as the decay time depends on the reading beam intensity. At low to moderate light intensities, the response time of the PRC is inversely proportional to the impinging intensity (the dielectric relaxation time is inversely proportional to the light intensity^21^), and at high intensities the response time is ultimately limited by the shortest recombination time of the charge carriers (to be discussed below). As a result, the hologram decay time drops with increasing light intensity and may fall below the pulse duration, resulting in a significant reduction in the overall diffraction efficiency.

The temporal response of BSO crystals can be explained by a band transport model (Fig. 2a)^23^,^24^. The existence of a shallow trap with a short recombination time *τ_R_* ≈ 4 ns gives rise to a fast carrier density decay at a moderate charge mobility value *μ* ≈ 0.25 cm^2^ V^−1^ s^−1^. In addition, due to the thermal excitation of the shallow traps and recombination associated with the deep traps, the response follows a double-exponential decay with another decay constant *τ_R_*′ ≈ 500 μs. As the reading beam intensity increases, the contribution of the shallow traps becomes dominant, resulting in a fast response^23^. For reading pulses with ~10 ns temporal width (Brilliant B laser), such an effect became clearly observable and affected the achievable gain significantly. In a first experiment, the energy of the R* beam (*E*_R*_) was increased gradually from 10 to 200 μJ, and a proportional increase of the S* beam energy (*E*_S*_) was observed (Fig. 2b), indicating a constant *η*. During the experiment, we measured *E*_R*_ by a power meter (S302C, Thorlabs; the R* repetition rate was 10 Hz) and *E*_S*_ by an energy meter (Vega, Ophir). We then further increased the reading pulse energy in eight steps up to 32 mJ, and observed that *η* began to decrease once *E*_R*_ reached 1 mJ, and finally dropped below 15% of its original value. The drop of the diffraction efficiency resulted in an energy gain clamped at around 2.3 × 10^−4^. The measurement results are plotted in Fig. 2c, where the left ***y***-axis is normalized against the value of *η* at *E*_R*_ = 200 μJ.

We then modeled the kinetics of the photorefractive effect by a set of coupled equations, taking into account the interaction among the charge carriers, shallow traps, deep centers, space charge field and the pumping radiation. The model employed to simulate the PRC transients is adopted and modified from Ref. 20. The band diagram, shown in Fig. 2a, illustrates a shallow and a deep trap level. Based on the hologram decomposition principle introduced in the preceding section, we consider only the transients involved in the readout of a sinusoidal hologram with grating vector **K**. All variables are separable into two parts: a background space-independent part, denoted by subscript “0”, and a spatially periodic part, denoted by subscript “1”. Both are time-variant (*t* denotes time):
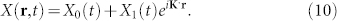


The zeroth order solutions for the carrier density *n*_0_ and the ionized donor density 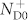 can be determined from
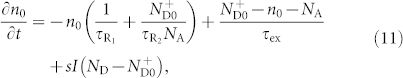

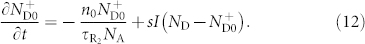
In the above equations, 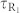,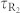 and *τ*_ex_ are the recombination time constants of the shallow and deep traps, and the thermal excitation time constant of the shallow traps, respectively (see Fig. 2a). *N*_A_ denotes the density of the ionized shallow acceptors, *s* denotes the photoionization coefficient, and *I* is the time-varying optical pulse intensity. The solutions to Eqs. (11) and (12) are inserted into the following equations to solve the first order dynamics:
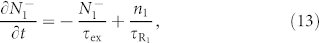

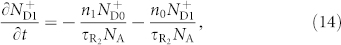

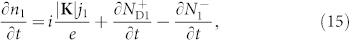

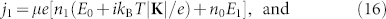

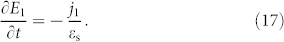
In the above equations, *N*^−^ denotes the density of occupied shallow traps, *j* denotes current, and *E* represents the space charge field.

We used *ε*_s_ = 56*ε*_0_ (*ε*_0_ is the vacuum permittivity), 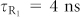, 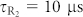, *τ*_ex_ = 200 ns, *μ* = 0.5 cm^2^ V^−1^ s^−1^, *N*_D_ = 10^19^ cm^−3^, *N*_A_ = 0.8 × 10^16^ cm^−3^, *s* = 0.2 *J* cm^2^, and |**K**| = 2*π*/Λ where Λ = *λ*/(2 sin *θ*), *λ* = 532 nm, *θ* = 13° in the simulation. The initial values of the first order terms are determined by the static solutions of the dynamic equations under CW illumination. The normalized instantaneous diffraction efficiency is found by the square of the SCF modulation magnitude normalized by its initial value: *η*_instant_(*t*) = |*E*_1_(*t*)/*E*_1_(0)|^2^. The total diffraction efficiency is calculated by
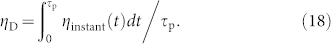
The numerical solution, assuming rectangular 10 ns pulses, is co-plotted in Fig. 2c for comparison. The agreement between the theory and the experiment is satisfactory.

We further increased *G*_p_ by shortening the duration of the R* pulse. On the one hand, when the time span of R* is reduced below the shortest decay constant of the crystal (4 ns), the entire pulse can be efficiently diffracted; on the other hand, squeezing the duration of the reading pulse increases the peak power if the pulse energy is conserved. Accordingly, we employed a picosecond laser (APL-4000, Attodyne) as the source for R*. The scattering medium, shown in Fig. 3a, is composed of a cuvette filled with water and placed between two parallel ground glass diffusers (DG10-600, Thorlabs), separated by 3 cm. We focused light inside two scattering layers rather than inside a bulk scattering medium (such as an intralipid phantom) to have convenient access to the TRUE focus. To justify the turbidity of the scattering sample, we measured the optical thickness^2^ of a single diffuser to be 14 at 532 nm (the fraction of the ballistic light through one diffuser is below 1 × 10^−6^). Focusing between two scattering layers is analogous to focusing inside a bulk scattering medium in that the target position is inaccessible externally^25^. A 50:50 beamsplitter (a 0.5 inch cube) was placed inside the cuvette to produce a copy of the TRUE focus (point “1” in Fig. 3a), which was further imaged by a doublet lens (*f* = 70 mm) onto a complementary metal-oxide semiconductor (CMOS) camera (Firefly MV, Point Grey) with unity magnification (point “2” in Fig. 3a). To record a hologram, part of the light passing though the ultrasonic focus was tagged with a frequency shift due to the acousto-optic effect. The field of the tagged light was holographically recorded and phase-conjugated to induce focusing at the ultrasonic focus. In the experiment, 7.5 MHz focused ultrasound was applied perpendicularly to the optical axis by an immersion transducer with a numerical aperture of 0.25 (Olympus). The distance between the back surface of the first diffuser and the ultrasound focus was 1 cm. The light beam impinging on the first diffuser was 1 mm in diameter. We set *f*_1_ = 50 MHz and *f*_2_ = 57.5 MHz, so that the frequency-down-shifted light emanating from the ultrasonic focus, when interfering with R, contributed to a stable hologram. The image of the TRUE focus is shown in Fig. 3b. The focus disappeared when *f*_2_ was offset by 100 kHz (in this case no hologram was recorded). The lateral full width at half maximum (FWHM) of the focus was 810 μm, almost twice the size of the theoretically predicted value (410 μm). The broadening is tentatively attributed to aberration caused by misalignment of the imaging system, and to an insufficient acceptance angle of the lens. The horizontal interference pattern superimposed on the focus is supposedly a side effect from the ultrasonic modulation, but the actual reason is still unclear. We adjusted the ratio between the S and R beams so as to maximize the focal intensity. Under the optimal beam ratio, we estimated *P*_s_ by putting a 1 mm diameter iris at a distance of 1 cm behind the first diffuser (to replace the ultrasound focus), and measuring the power through the iris (using a Thorlabs S302C power meter; measurements were performed in air). *P*_s_ was measured to be 1.5 mW, and *E*_R*_ was 40 μJ per pulse in the experiment.

We used a Twyman-Green interferometer to measure the temporal profile of the 532 nm output from the laser, as shown in Fig. 3c. The dashed lines denote a pair of planes, symmetric with respect to the beam-splitting plane. Mirror M1 moves perpendicularly to the respective beam path to generate a global delay of 2Δ*L*/*c*, whereas mirror M2 is fixed but tilted at a small angle *θ*/2 so that the two beams (both are plane waves) interfere on the CMOS detector plane at an angle *θ*, which converts to a position-dependent optical pathlength difference (OPD) on the camera, *δOPD*(*x*) ≈ *θx*.

On the camera, the two beams reflected by M1 and M2 are written as
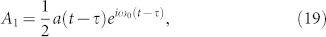

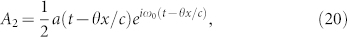
respectively, where *a*(*t*) is a slowly varying envelope of the electric field, *τ* = 2Δ*L*/*c* is the delay generated by M1, *c* is the light velocity, and *ω*_0_ is the central light frequency.

One may find that, after mathematical manipulations, the fringe seen on the camera (shown in Fig. 3d) can be expressed as
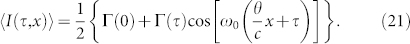
Here, the angle brackets denote time averaging, and the electric field autocorrelation function Γ(*τ*) is defined as
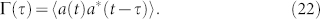
In arriving at Eq. (21), we assume that *θx*/*c* is negligible compared to the sampling interval of the time delay (Δ*τ*). We further assume that the pulse is chirp-free so that Γ(*τ*) is real^26^. From Eq. (21), we conclude that Γ(*τ*) can be inferred from the AC amplitude of the interferogram, which is shown in Fig. 3e (averaged over rows), and can be estimated by a fast Fourier transform (shown in Fig. 3f). The measured Γ(*τ*) is shown in Fig. 3g as discrete points with superimposed error bars; the dash-dotted line is the cubic spline interpolation of the data.

We assumed that the pulse shape is an even function of time, as implied from the pulse shape at 1064 nm (according to laser specification data provided by Attodyne Inc., Canada), so that it is transform limited (having a constant spectral phase). We then calculated the power spectrum by 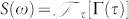, where 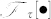 denotes the Fourier transformation. From 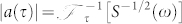, we obtained the field temporal profile (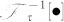 denotes the inverse Fourier transformation), shown in Fig. 3g as the dashed curve. The pulse shape was calculated from *I*(*τ*)∝|*a*(*τ*)|^2^, shown in Fig. 3g as a solid curve. The FWHM of the pulse, estimated from its temporal profile, is 3.5 ps.

The pulse is broadened upon reaching the TRUE focus. Given the dispersion properties and the total thickness of the dispersive materials along the optical path (~4 inches), we found the cumulative pulse broadening due to dispersion to be much less than the pulse duration. Two lenses, in a 4-*f* arrangement, image the back surface of the scattering sample, denoted as Plane A, onto the PRC entrance face with a magnification of ~3. The pulse remains a plane wave before passing through the PRC, and all rays diffracted by the PRC assume an identical OPD when reaching Plane A, according to the aforementioned image relationship. Consequently, we conclude that the time-reversed light impinging on the scattering medium is roughly 3.5 ps in duration. From the image relationship, the illuminated region on Plane A that effectively contributes to the TRUE focus has a dimension of ~10 (mm)/3 ≈ 3 mm (10 mm is the dimension of the BSO crystal, 3 is the magnification of the imaging system). The distance between Plane A to the TRUE focus is ~2 cm. Given these numbers, a simple geometric calculation yields a maximum OPD difference, among the rays traveling from Plane A to the TRUE focus, of ~0.1 mm, amounting to a broadening of 0.3 ps. We used a duration of 5 ps as a conservative estimate.

To characterize the power gain, the camera in Fig. 3a was replaced by an energy meter (Vega, Ophir). *P*_TR_ was estimated by dividing the measured pulse energy by its duration (5 ps). The power gain was characterized at various hologram writing times (*T*_w_) and fitted by^18^ *G*_p_ = *a*[1 − exp(−*T*_w_/*b*)] (*a* and *b* are the fitting parameters). The fitting yields a rise time of 0.37 s defined at (1 − e^–1^) of the full grating strength. The experimental results, along with the fitting curve, are plotted in Fig. 3h. The maximum power gain attained was 33,000 times (45 dB), with a focal light intensity exceeding 6400 W cm^−2^ (estimated by a circular focal area with 1 mm diameter).

In the above TRUE focusing experiment, the energy gain was estimated to be ~0.3 × 10^−6^, much less than that obtained in the “direct transmission” experiment (2.3 × 10^−4^). Several factors contribute to the reduction: the hologram is weaker (<10% in strength) because the untagged light contributes to a raised background; the scattering sample with higher turbidity renders light collection less efficient, reducing *η*_1_; and finally, the energy of the reading pulse was smaller. For a picosecond readout, the achieved power gain will reduce at increased sample turbidity, as a result of the temporal broadening associated with the increased photon path length variance^27^. Equivalently, a larger path length variance demands for greater laser coherence, leading to the requirement of wider reading pulses and consequently less power gain at the same pulse energy. The response time of 0.37 s is more than two orders of magnitude larger than the tissue correlation time (1 ms). This problem can be overcome by increasing the power of S and R to decrease the dielectric relaxation time^18^. The low reading pulse energy used in the experiment (40 μJ/pulse) implies that the gain has great potential to grow. It should be noted that Eq. (1) holds only when the photodetector has a sufficient bandwidth to recover the pulse shape. Accordingly, a picosecond pulse is too short to increase the SNR of a linear imaging system. One may instead use an intense sub-nanosecond pulse to ensure high diffraction efficiency and a bandwidth compatible with fast photodetectors (photodiodes with 10 GHz bandwidth are commercially available). The power gain is restricted by the optical limiting effect stemming from excited state absorption and two-photon absorption, and is ultimately limited by the damage threshold of the crystal (1.5 GW cm^−2^ for nanosecond pulses and 30 GW cm^−2^ for picosecond pulses)^28^,^29^. For nonlinear applications demanding very high intensities, TRUE focusing in its original form may become incompetent when the medium's turbidity is high, since the multiple scattering process tends to broaden the pulse duration, and a spatio-temporal focusing is required^30^,^31^.

In summary, we studied the efficiency and dynamics of analog TRUE optical focusing and demonstrated a hybrid CW/pulsed system to overcome the inherent low energy conversion efficiency. An unprecedented power gain of 33,000 times at 532 nm was obtained between two scattering layers. This study is an important step toward real-world implementations of TRUE focusing, enabling a number of applications in optical imaging, sensing, manipulation and therapy inside scattering media.

## Methods

### System set-up and timing

The system set-up is shown in Supplementary Fig. S1. The RF and trigger signals share the same time base. An isolator IS (Thorlabs IO-5-532-HP) is inserted in the R path to protect the continuous wave (CW) laser. For the TRUE focusing experiment, the RF frequencies are 57.5 MHz, 50 MHz, 50 MHz, and 7.5 MHz for RF1 to RF4, respectively. The externally applied field EF has a square waveform with 2 kHz rep rate and 6 kVpp amplitude. Each focusing cycle, lasting 2 seconds, encompasses three steps. In the hologram writing step, the shutter ST is open and acousto-optic modulator AOM3 is idle. In the hologram reading step, ST is closed and AOM3 is fired for 0.9 ms; for the 1 kHz repetition rate of R*, this timing allows the hologram to be read by a single pulse. At the same time, the CMOS camera is triggered to image the TRUE focus. In the hologram erasing step, ST is closed and AOM3 is fired for 0.5 s to clear the residual hologram with 500 identical pulses. The inset of Fig. S1 shows the TTL signals Tr1 and Tr3, applied to control the hologram writing, reading and erasing procedures. Tr3 controls hologram writing, with an adjustable duration. The short initial spike in Tr1 is the reading control, which allows only a single pulse to read the hologram. The ensuing longer pulse in Tr1 ensures that the hologram is erased by multiple reading pulses.

## Author Contributions

C.M. and L.V.W. initiated the project. C.M. and X.X. implemented the system. C.M. ran the experiments, performed the simulation, and processed the experimental results. L.V.W. provided overall supervision. All authors involved in writing the manuscript.

## Supplementary Material

Supplementary Information Supplementary note and figure

## Figures and Tables

**Figure 1:**
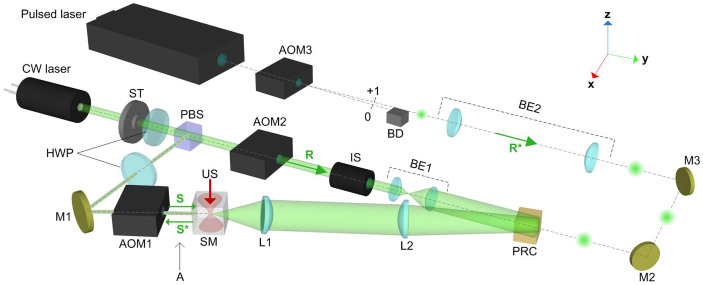
Experimental set-up (see text for details). AOM, acousto-optic modulator; BD, beam dump; BE, beam expander; HWP, half-wave plate; IS, optical isolator; L, lens; M, mirror; PBS, polarizing beamsplitter; PRC, photorefractive crystal; SM, scattering medium, shown enlarged in Fig. 3a; ST, optical shutter; US, ultrasound; point A, the location where S* is assessed in the “transmission through” experiment.

**Figure 2:**
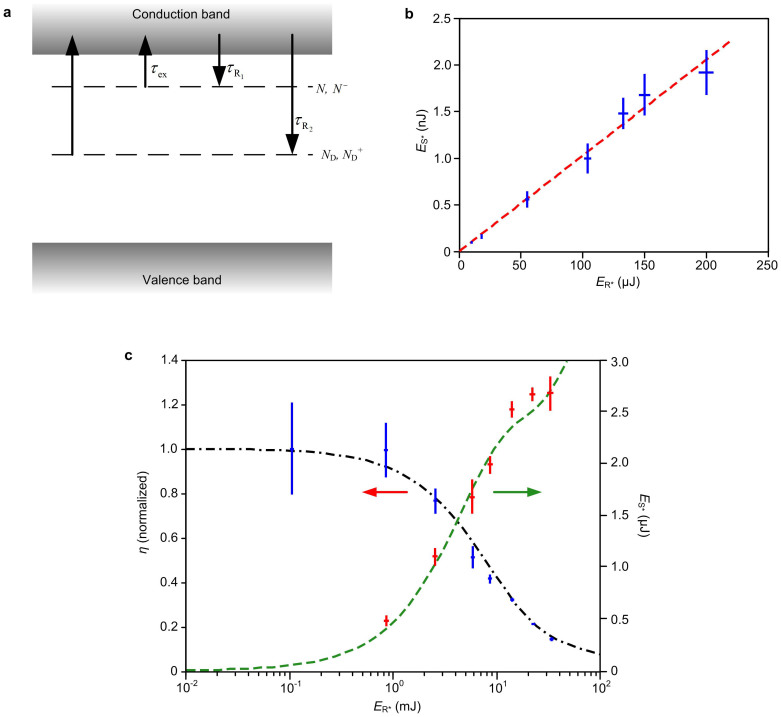
Results of the “transmission through” experiment with nanosecond laser readout. (a) Band diagram of the BSO model. (b) Conjugate versus reading pulse energy. The linear fitting (in dashed line) shows an effective diffraction efficiency of ~10^−5^. (c) Normalized diffraction efficiency and the conjugate beam energy plotted against the reading pulse energy. Dash-dotted and dashed curves are numerical results from the model. Vertical error bars represent the standard deviations of 20 measurements. Standard errors are not plotted due to their undiscernible lengths in the figure. Horizontal error bars are generated based on the accuracy of the power meter.

**Figure 3:**
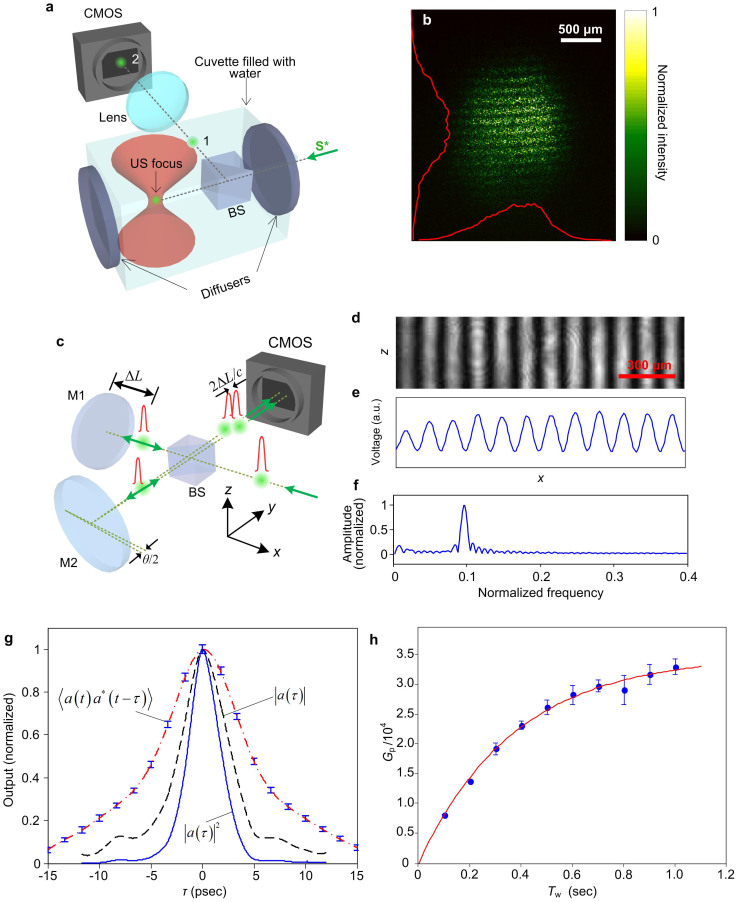
Results of the TRUE focusing experiment with picosecond laser readout. (a) Arrangement of the phantom and the focus visualization scheme. BS, non-polarizing beam-splitter. (b) The image of the TRUE focus. The horizontal and vertical profiles of the focus (after two-dimensional smoothing) are co-plotted as red solid curves. (c) Set-up for measurement of the pulse duration. (d) Fringe pattern on the CMOS camera. (e) Fringe intensity along *x*. (f) Fourier transformation of (e). (g) Pulse shape measurement results. Error bars represent the standard deviation (SD), and standard errors are not plotted due to their undiscernible lengths in the figure. The mean and SD are estimated based on 10 measurements. (h) Measured gain as a function of the hologram writing time (*T*_w_). Error bars represent the standard deviation of 20 measurements, and standard errors are not plotted due to their undiscernible lengths in the figure.
