# Supplementary material for: Analog time-reversed ultrasonically encoded light focusing inside scattering media with a 33,000× optical power gain
Source: Sci Rep. 2015 Mar 10;5:8896. doi: 10.1038/srep08896 (PMC4354154; doi:10.1038/srep08896)
Supplement: Supplementary Information — Supplementary note and figure [file srep08896-s1.pdf]

Analog time-reversed ultrasonically encoded light focusing inside scattering media with a 33,000× optical power gain

Cheng Ma, Xiao Xu and Lihong V. Wang*

Optical Imaging Laboratory, Department of Biomedical Engineering,

Washington University in St. Louis, St. Louis, Missouri 63130-4899

*Corresponding author: lhwang@wustl.edu

Supplementary note

Signal-to-noise ratio (SNR) of optical detection

If the incident optical illumination generates an average photocurrent $\overline{i_{\mathrm{s}}}$, the amplitude SNR of the photodetector output is[29]

$$SNR = \frac{\overline{i_{\mathrm{s}}}}{\sqrt{\left[2e\overline{i_{\mathrm{s}}} + 2e\overline{i_{\mathrm{b}}} + \frac{4k_{\mathrm{B}}T}{R_{\mathrm{L}}}\right]\Delta\nu}}, \tag{S1}$$

where e is the elementary charge, $\overline{i_{\mathrm{b}}}$ is the average reverse bias leakage current, k_{B} is the Boltzmann constant, T is the absolute temperature, R_{L} is the load resistance and $\Delta\nu$ is the bandwidth of the detector. The three terms on the denominator account for the signal shot noise, dark-current shot noise, and thermal noise.

A pulse with a duration τ_{p} has a bandwidth of $\Delta\nu \approx 1/\left(2\tau_{\mathrm{p}}\right)$. Denoting the pulse energy as E_{p}, the bandwidth can be expressed in terms of E_{p} as

$$\Delta\nu = \frac{\overline{i_{\mathrm{s}}}}{2E_{\mathrm{p}}\rho_{\mathrm{d}}}, \tag{S2}$$

where ρ_d denotes the photodetector's responsivity. Inserting Eq. (S2) in (S1) we get

$$SNR = \sqrt{\frac{E_\mathrm{p}}{\frac{\alpha}{\overline{P}} + \beta}} \ . \qquad \text{(S3)}$$

In Eq. (S3), $\alpha = \left(e\overline{i_\mathrm{b}} + 2k_\mathrm{B}T / R_\mathrm{L}\right) \big/ \rho_\mathrm{d}^2$ and $\beta = e / \rho_\mathrm{d}$ are constants, and $\overline{P}$ denotes the average power of the pulse ($E_\mathrm{p} \big/ \tau_\mathrm{p}$).

Reference

29 Yariv, A. & Yeh, P. *Photonics: Optical Electronics in Modern Communications (The Oxford Series in Electrical and Computer Engineering).* (Oxford University Press, Inc., 2006).

Supplementary figure

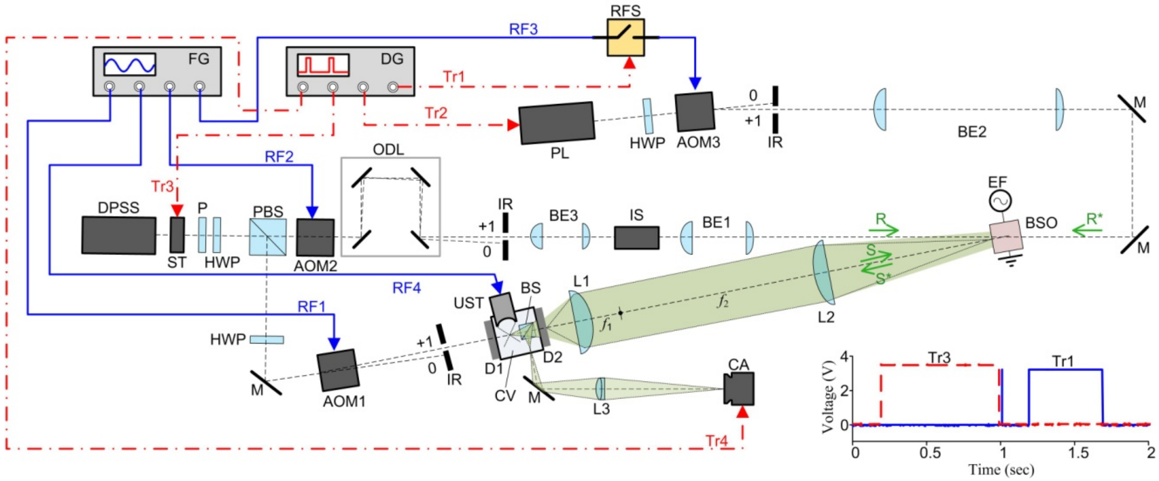

Figure S1. System setup. AOM, acousto-optic modulator; BE, beam expander; BS, 50:50 non-polarizing beamsplitter; BSO, $Bi_{12}SiO_{20}$ crystal; CA, CMOS camera or photodiode; CV, water-containing cuvette; D, diffuser; DG, delay generator; DPSS, diode-pumped solid-state laser; EF, externally applied field; FG, function generator; HWP, half-wave plate; IR, iris; IS, optical isolator; L, lens; M, mirror; ODL, optical delay line; P, optical polarizer; PBS, polarizing beamsplitter; PL, pulsed laser; RF, radio-frequency driving signal; RFS, radio-frequency switch; ST, optical shutter; Tr, trigger signal; UST, ultrasonic transducer. Inset: oscilloscope traces of Tr1 and Tr3.
